# The RICO dataset: A multivariate HVAC indoors and outdoors time-series dataset

**DOI:** 10.1016/j.dib.2025.111678

**Published:** 2025-05-18

**Authors:** Zachari Thiry, Massimiliano Ruocco, Alessandro Nocente, Odne Andreas Oksavik

**Affiliations:** a SINTEF Manufacturing, SP Andersens Veg 3, Trondheim, Norway; b SINTEF Digital, Strindvegen 4, Trondheim, Norway; c SINTEF Community, Høyskoleringen 7B, Trondheim, Norway

**Keywords:** Forecasting, Machine learning, Temperature, Energy, Environment control

## Abstract

Indoor temperature forecasting is an area of interest and importance as it contributes to improving HVAC systems control, thus reducing wasted energy and improving health and comfort. Acquiring high-quality transitory regime data for training Machine Learning models is challenging due to the scarcity of publicly available dataset. Additionally, such a dataset acquisition incurs high costs from repeated heating and cooling buildings in ranges of temperatures that go beyond normal operation thresholds. In response, we propose an open-source dataset called ‘RICO Dataset’. It is acquired in a dedicated and controlled physical test-building, alleviating potential issues encountered by digital simulation and modelling. It contains 305, four hours long 80-features rich multivariate transitory time series data from sensors in both internal and external environments sampled at a rate of one per minute.

Specifications Table

**Table:** 

Subject	Computer Sciences
Specific subject area	Indoors and outdoors environmental sensors time-series of transitionary regimes
Type of data	Table (hdf format) - Timestamp index - Integers - Float numbers It includes joined sensor data and acquisition metadata in single table.
Data collection	The dataset comprises high sampling rate measurements from sensors of a physical test building, collected as part of a Machine Learning experiment [1]. Two sensor types are utilized: internal sensors measuring temperature, humidity, and radiation, and external sensors from the building's dedicated weather station. The data points were collected across five distinct phases, lasting from a few days to 17 days over a period of 11 months, capturing diverse weather conditions. This resulted in 305 multivariate time-series, each four hours long and 80 features rich. All measurements were recorded at 10 samples per minute and normalized to a 1-min sampling interval for consistency.
Data source location	Trondheim, Norway, in the ZEB Test Cell Laboratory - SINTEF Coordinates of Acquisition: 63.41441150601661, 10.409144324548096
Data accessibility	Repository name: data.sintef.no [2] Data identification number: 10.60609/TW79-4K72 Direct URL to data: https://zenodo.org/records/14871584 Instructions for accessing the data: 1. Login: Click on the provided link and follow the data URL to access the Zenodo platform. 2. Scroll down the main section past the README file. 3. Download files utilizing the “Download all” button on the top right of the files table.
Related research article	*Z. Thiry, M. Ruocco, A. Nocente, M- Spitieris, Enhancing Indoor Temperature Forecasting through Synthetic Data in Low-Data Environments (2024)* [1] 10.3384/ecp208

## 1 Value of the Data

• The RICO dataset captures constrained multivariate transitory regimes within indoor temperature environments, typically absent from existing operational datasets due to the costs generated by successively heating and cooling a facility. This inclusion allows researchers to explore complex dynamics between several variables and is it suitable for studying and training models on transitory regimes.
• The RICO dataset includes comprehensive multivariate data collected from both indoor sensors and a dedicated external weather station. This approach ensures that indoor temperature variations can be analysed in relation to external environmental factors, such as outdoor temperature, humidity, and other weather conditions. These features allow researchers to study the influence of external factors on indoor climate control systems, which can affect the accuracy of predictive models. Additionally, it provides support for the development of context and correlation aware systems and machine learning models.
• The RICO Dataset has been acquired over varying weather conditions and seasons. By including data that encompasses different seasonal and weather contexts, this dataset allows researchers to study and build models that generalize across a wide range of conditions.
• The RICO dataset can serve as a benchmark for comparing the performance of different machine learning approaches. Researchers can reuse it to train and validate new predictive models, thus contributing to advancements in HVAC control systems, and more generally to the field of Machine Learning.

## 2 Background

The RICO dataset was compiled to address the need for high-quality, real-world data in the field of indoor temperature forecasting, a fundamental aspect of building management systems. Predictive models for HVAC systems, designed to enhance energy efficiency and occupant comfort, have gained in popularity [[3], [4], [5]], but remain constrained by the scarcity of comprehensive datasets. Previous studies [3,6,7] have frequently resorted to synthetic data or limited datasets, particularly in the context of neural-based approaches to system control. Additionally, Afram et al. [8] highlight challenges in data accuracy, sampling rates, and the lack of environmental information.

The uniqueness of this dataset stems from its focus on transitory regimes of HVAC systems, a unique aspect not covered by other datasets. Additionally, it includes weather data from an on-siter weather station, offering more precise, and relevant information compared to datasets relying on external sources. The dataset was developed in a controlled environment at the ZEB TEST Cell laboratory [9], ensuring a high sampling rate and verification of the data. By offering this dataset, we seek to contribute valuable real-world data to facilitate further research and model development in HVAC, energy efficiency, and time-series forecasting.

## 3 Data Description

The RICO dataset consists of a set of five files named *RICO_Acquisition_<X>_MM-YYYY.hdf*, where *<X>* denotes the acquisition phase number, *MM* and *YYYY* the respectively the month and year of acquisition, saved into a h5 format. Each file contains multi-variate time-series of measurements from both the indoor controlled environment of the test facility, and the outdoor weather conditions registered by a dedicated weather station. All files are stored in a long format, meaning that rows are indexed by a timestamp. Depending on the acquisition phase (see next section), the dataset contains either 81 or 82 features. The feature types can be grouped as such:

- Metadata features: such as Phase, Step, Flag, and control points.
- Observable sensors: temperatures, humidity, wind-speed.
- Operational information: such as pumps or ventilators status and HVAC control sensors.

**Fig. 1:**
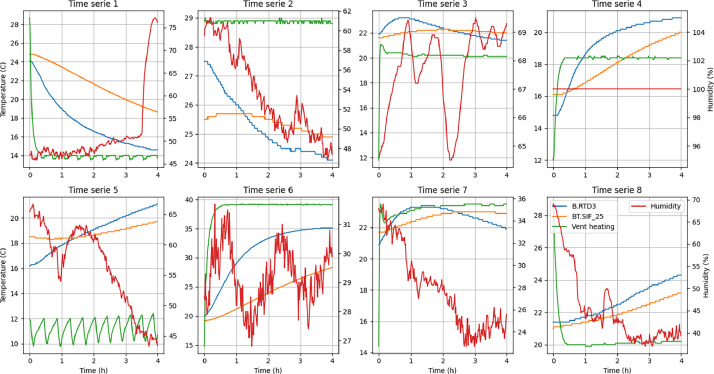
Dataset samples representation, four selected features only.

A complete list of all the features and their description is given in Table 2. Additionally, a simplified representation of a dataset sample in Table 1, and a visualization of some time series in Fig. 1 displayed below.

**Table 1:** Simplified Dataset Samples: This table presents one-minute average readings from the dataset, with feature names and values simplified for readability and understandability. Each row includes a timestamp (Time), metadata (Phase, Step, Flag), control points (EC3, SB43, SB46, SB47), operational information (e.g., JP40), and observed sensor values (BT.7, Pressure, Dew Point, Humidity, Wind Direction, and others). For a complete list of available features, refer to Table 2.

Time	Phase	Step	Flag	EC3	SB43	SB46	SB47	BT.7	JP40	Pressure	Dew Point	Humidity	Wind Direction
14:59	1	0	0	30.0	0.0	7.0	20.0	29.8	0.8	992.4	13.2	63.4	136.7
15:00	1	0	0	30.0	0.0	7.0	20.0	29.8	0.8	992.4	13.6	63.1	27.2
15:01	1	1	1	40.0	0.0	0.0	40.0	29.8	0.3	992.4	13.4	62.7	183.0
15:02	1	1	1	40.0	0.0	0.0	40.0	29.9	0.0	992.4	13.2	62.6	107.0
15:03	1	1	1	40.0	0.0	0.0	40.0	30.2	0.0	992.4	13.3	62.9	176.7

**Table 2:** Complete Features List. The table contains names, measured quantities, units and a short description of each feature. The features are grouped in relevant subgroups: first, the metadata and control points; the operational information sensors for each of the four actuators; and finally, the list of observable variables.

Name	Measured Quantity	Units	Description
Metadata			
Flag		-	1 if the quality is low, 0 else
Scheduler Step		-	Step in the data acquisition schedule
Acquisition Phase		-	Specific phase number of data acquisition
Radiator Heating			
SB47	Valve Flow	%	Radiator Heater circuit valve – Value
pid.SB47.setpoint	Setpoint	°C	Radiator Heater circuit valve – Value
pid.SB47.enabled	On/Off	Bool	Radiator Heater Toggle
JP41_setpoint	Setpoint	%	Main Hot Pump setpoint
JP41_volumeFlow		m^3^.h^-1^	Main Hot Pump volume flow
JP41_head		m	Main Hot Pump head
JP410_setpoint	Setpoint	%	Radiator Specific Pump
JP410_volumeFlow		m^3^.h^-1^	Radiator Specific Pump volume flow
JP410_head		m	Radiator Specific Pump head
RTD420	Temperature	°C	Radiator Heater Inlet water temperature
RTD509	Temperature	°C	Radiator Heater Outlet water temperature
Fan Coil			
SB46	Valve Flow	%	Radiator Heater circuit valve – Value
pid.SB46.setpoint	Setpoint	°C	Radiator Heater circuit valve – Value
pid.SB46.enabled	On/Off	Bool	Radiator Heater Toggle
JP40_setpoint	Setpoint	%	Main Hot Pump setpoint
JP40_volumeFlow		m^3^.h^-1^	Main Hot Pump volume flow
JP40_head		m	Main Hot Pump head
JP49_setpoint	Setpoint	%	Radiator Specific Pump
JP49_volumeFlow		m^3^.h^-1^	Radiator Specific Pump volume flow
JP49_head		m	Radiator Specific Pump head
RTD417	Temperature	°C	Radiator Heater Inlet water temperature
RTD508	Temperature	°C	Radiator Cooler Outlet water temperature
Ventilation Heating			
EC3	Power	%	Ventilation Header Cell B
pid.EC3.setpoint	Setpoint	°C	Ventilation Heater Setpoint
Pid.EC3.enabled	On/Off	Bool	Ventilation Heater Toggle
RTD410.T	Temperature	°C	Ventilation Heater Inlet Air Temperature
RTD406A	Temperature	°C	Ventilation Heater Inlet Air Temperature
B.ASTRHT2.T	Temperature	°C	Ventilation Duct Temperature Entering Room
Ventilation Cooling			
SB43	Valve Flow	%	Ventilation Cooler Valve
pid.SB43.setpoint	Setpoint	°C	Ventilation Cooler Setpoint
pid.SB43.enabled	On/Off	Bool	Ventilation Cooler Toggle
JP40_setpoint	Setpoint	%	Main Hot Pump setpoint
JP40_volumeFlow		m^3^.h^-1^	Main Hot Pump volume flow
JP40_head		m	Main Hot Pump head
JP44_setpoint	Setpoint	%	Specific Cooling Pump setpoint
JP44_volumeFlow		m^3^.h^-1^	Specific Cooling Pump volume flow
JP44_head		m	Specific Cooling Pump head
RTD410.T	Temperature	°C	Ventilation Cooler Inlet Air Temperature
RTD406A	Temperature	°C	Ventilation Heater Inlet Air Temperature
B.ASTRHT2.T	Temperature	°C	Ventilation Duct Temperature Entering Room
Observation sensors			
B.ASTRHT2.S	Air Speed	m.s^-1^	Ventilation Duct Outlet Air Speed
B.ASTRHT2.H	Humidity	%	Ventilation Duct Outlet Humidity
B.RTD1	Temperature	°C	Room Temperature – Centre – 10cm height
B.RTD2	Temperature	°C	Room Temperature – Centre – 50cm height
B.RTD3	Temperature	°C	Room Temperature – Centre – 100cm height
B.RTD6	Temperature	°C	Room Temperature – Centre – 150cm height
BT13	Temperature	°C	Window Inside Temperature (no radiation)
BT15	Temperature	°C	Room Temperature – Centre – 180 cm
BT.AON	Temperature	°C	Temperature Air Outside North
BT.AOE	Temperature	°C	Temperature Air Outside East
BT.AOW	Temperature	°C	Temperature Air Outside West
BT.AOF	Temperature	°C	Temperature Air Outside Floor
BT.AOC	Temperature	°C	Temperature Air Outside Ceiling
BT.SON	Temperature	°C	Temperature Surface Outside North
BT.SOE	Temperature	°C	Temperature Surface Outside East
BT.SOW	Temperature	°C	Temperature Surface Outside West
BT.SOF	Temperature	°C	Temperature Surface Outside Floor
BT.SOC	Temperature	°C	Temperature Surface Outside Ceiling
BT.SIC_25	Temperature	°C	Temperature Surface Inside Ceiling Door
BT.SIC_26	Temperature	°C	Temperature Surface Inside Ceiling Centre
BT.SIC_27	Temperature	°C	Temperature Surface Inside Ceiling Window
BT.SIE_7	Temperature	°C	Temperature Surface Inside East Door
BT.SIE_8	Temperature	°C	Temperature Surface Inside East Centre
BT.SIE_9	Temperature	°C	Temperature Surface Inside East Window
BT.SIF_25	Temperature	°C	Temperature Surface Inside Floor Door
BT.SIF_26	Temperature	°C	Temperature Surface Inside Floor Centre
BT.SIF_27	Temperature	°C	Temperature Surface Inside Floor Window
BT.SIN_17	Temperature	°C	Temperature Surface Inside Unspecfied
BT.SIW_7	Temperature	°C	Temperature Surface Inside Window Left
BT.SIW_8	Temperature	°C	Temperature Surface Inside Window Centre
BT.SIW_9	Temperature	°C	Temperature Surface Inside Window Right
BF.SIM1	Radiation	W.m^-2^	Solar Radiation Behind Window
WS1_Absolute_humidity	Humidity	%	Absolute Humidity at Weather Station
WS1_Barometric_pressure	Pressure	hPa	Barometric Pressure at Weather Station
WS1_Dew_point	Temperature	°C	Dew Point at Weather Station
WS1_Relative_humidity	Humidity	%	Relative Humidity at Weather Station
WS1_Solar_radiation	Radiation	W.m^-2^	Solar Radiation at Weather Station
WS1_Temperature	Temperature	°C	Air Temperature at Weather Station
WS1_Wind_direction	Bearing	-	Wind Direction at Weather Station
WS1_Wind_speed	Speed	m.s-1	Wind Speed at Weather Station
O.SIM1	Radiation	W.m^-2^	Solar radiation on roof – slope ∼ 50°
O.SIM2	Radiation	W.m^-2^	Solar radiation on roof – vertical
O.SIM3	Radiation	W.m^-2^	Solar radiation on façade
JV41	Fan Speed	%	Main Ventilation Intake Fan
JV42	Fan Speed	%	Cell B Ventilation Intake Fan
JV51	Fan Speed	%	Main Ventilation Extraction Fan

## 4 Experimental Design, Materials and Methods

### 4.1 The acquisition facility

The following section describes the acquisition facility, also referred to as test facility, leveraged to acquire the RICO Dataset.

**Fig. 2:**
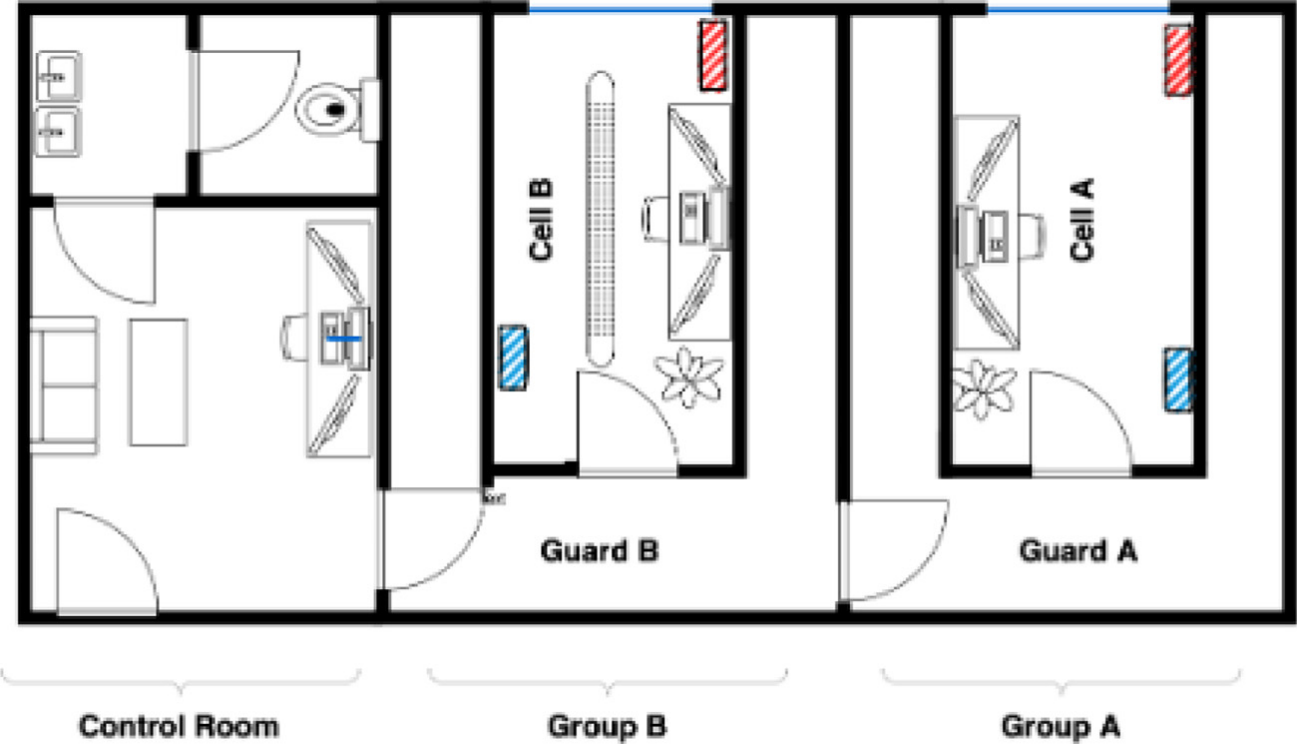
Test facility overview.

**Fig. 3:**
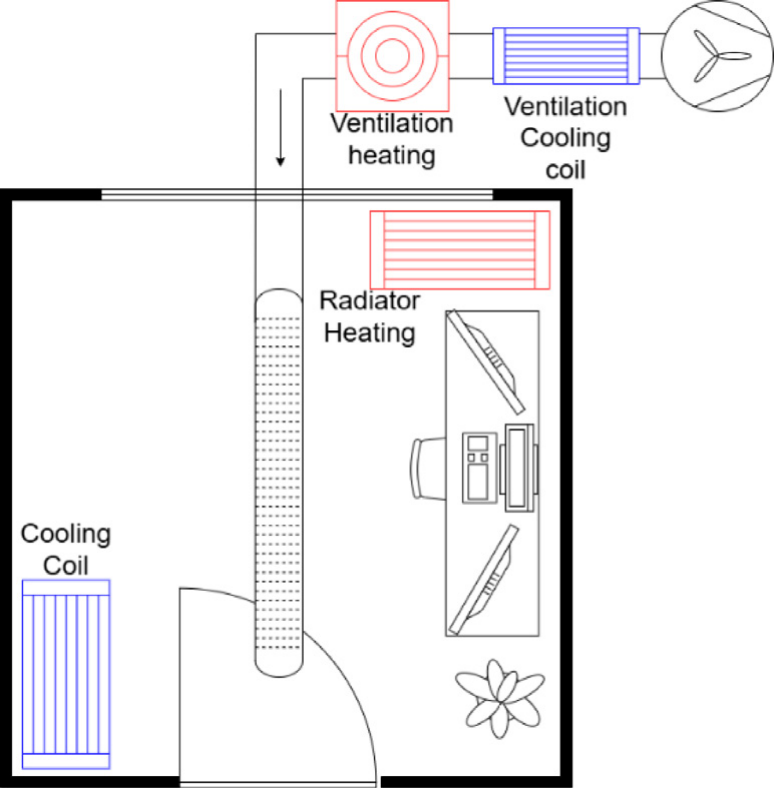
HVAC systems of cell B.

As illustrated in Fig. 2, the test facility contains two rooms for experimentation, denoted as Cell A and Cell B. Our focus is exclusively on Cell B, which houses the various HVAC systems shown in Fig. 3. First, a common ventilation intake duct is equipped with both an electric heater and a hydraulic heat exchanger cooler. Additionally, it houses two other water-based heat exchangers, each dedicated to either heating or cooling and running on separate water circuits. Finally, the test-cell is surrounded above, below and on three walls by guard room B whose air temperature is controlled and monitored to remain stable through the acquisition phases. The external wall has a window facing South, which allows for heat exchanges and radiation to enter the room.

### 4.2 Control of the HVAC system

The HVAC system is controlled using a Proportional-Integral-Derivative (PID) controller, a control loop feedback mechanism widely used in industrial control systems. The PID controller calculates an error value as the difference between a desired set-point and a measured process variable, and applies a correction based on proportional, integral, and derivative terms. Each of the four actuators of the HVAC system has their own PID controller, first approximately tuned using the Ziegler-Nichols method [10] and then fine-tuned manually. As such, each actuator is controlled by a different sensor, internal to its functioning:

• Ventilation heating (EC3): regulator - pid.EC3.setpoint & process value - B. ASTRHT2.T.
• Ventilation cooling (SB43): regulator – pid.SB43.setpoint & process value - RTD406A.
• Radiator heating (SB47): regulator – pid.SB47.setpoint & process value – RTD420.
• Radiator cooling (SB46): regulator – pid.SB46.setpoint & process value – RTD417.

**Fig. 4:**
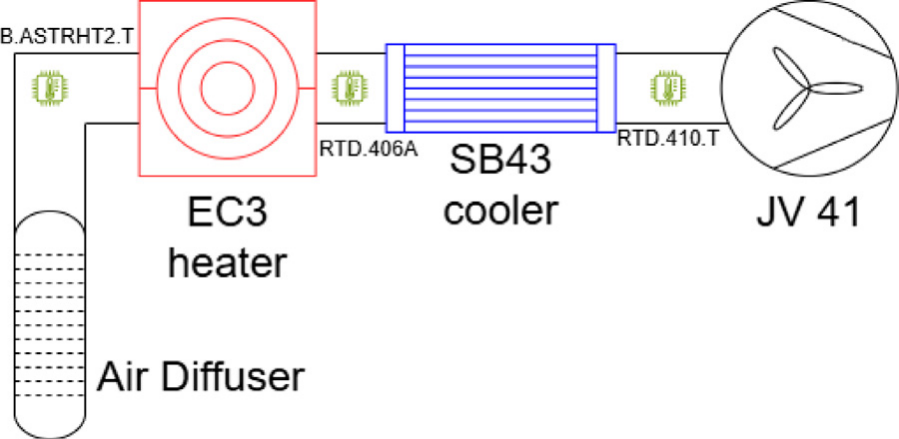
Ventilation Sensors and Actuators. Air is pumped externally through the JV 41 fan, then either cooled (SB43) or heated by the EC3 electric heater and finally diffused into the room through a tubular air diffuser.

**Fig. 5:**
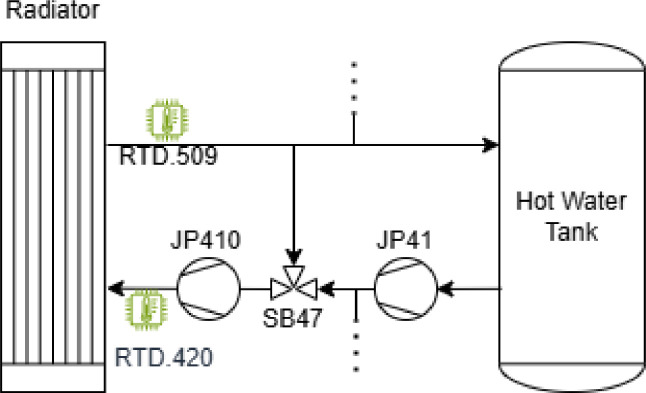
Heating System Schematic. The system controls the radiator heating device, is pressurised by two pumps (JP41 and JP410) and regulated by the proportional valve SB47. The dotted lines represent additional sub-systems that are not relevant for the experiment.

**Fig. 6:**
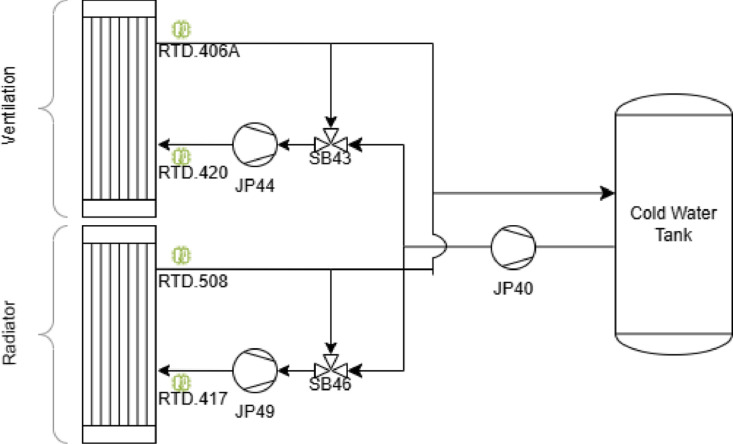
Cooling System Schematic. The cold-water circuit is shared between both cooling devices (radiator coil and ventilation coil). The main pump JP40 pressurises the entire circuit. Each sub-circuit is controlled by a proportional valve (SB43 or SB46) and has its own pressurising pump (JP44 and JP49). Intake and outflow water temperatures are measured by RTD sensors located at specific points within the circuit.

Some detailing figures pertaining to the different actuators and their hydraulic circuits are explained in Fig. 4, Fig. 5, Fig. 6.

### 4.3 Design of acquisition phases

To cover as many different weather conditions as possible whilst minimizing the energy use of the building and costs, it was decided to acquire the data over distinct phases of a few days spread out across a year.

The ‘*schedule’* is the core element that determines the acquisition parameters, such as timeseries length and setpoint combinations. A schedule consists of a series of steps, each being a four-hour period. The length of this period was evaluated to be roughly twice the characteristic response time of the test-cell which was evaluated experimentally. Each step is associated with specific control points, which are variables controlling the HVAC system. The control points are set to specific values at the beginning of each step and remain constant throughout the step. One step ultimately results in one series of data points. A simple python code generates a sequence of steps with randomized control points, thus producing a schedule.

The schedule is later run in the facility where the data is sampled raw at a frequency of 10 samples per minute, aggregated and averaged over 1-minute intervals to an apparent sampling rate of 1 sample per minute.

### 4.4 Specificities to each acquisition phase

The dataset acquisition process for the RICO Dataset was segmented into five distinct phases, each in a specific period ranging from July 2023 to May 2024, encompassing a total of 61 days’ worth of data. Table 3 provides a summary of the acquisition phases, including the number of data points collected, the duration of the acquisition period and the type of runs conducted. The column *Run Type* is organized as *(α + β) h* where *α* is the uptime duration in hours during which the HVAC system is ON within each step, and *β* is the downtime duration during which the HVAC system is OFF at the end of each step.

**Table 3:** Summary of the RICO Dataset acquisition phases.

Phase	Number of series	Start Date (Z)	End Date (Z)	Run Type
1	102	26/07/2023 13h	12/08/2023 13 h	(4+0) h
2	60	20/10/2023 13h	30/10/2023 13 h	(3+1) h
3	24	22/01/2024 14h	26/01/2024 14 h	(12+4) h
4	60	29/01/2024 16h	07/02/2024 16 h	(4+0) h
5	60	08/05/2024 08h	18/05/2024 08 h	(4+0) h

Additionally, acquisitions 3, 4, and 5 feature an additional feature recording the inside solar radiation sensor BF.SIM1, unrepresented in acquisitions 1 and 2.

## Limitations

We have identified two main limitations in this dataset. The first relates to data quality. In the initial data acquisition phase, the PID controllers for the HVAC system actuators were not fully optimized, leading to oscillations around the specified setpoints for all four actuators. This did not significantly impair the overall functioning of the HVAC system but lead to a different operation behaviour in the RICO_1 dataset compared to in subsequent acquisitions.

The second limitation concerns the consistency of run types. Experimental interests related to the project for which the data was acquired required that some downtime, or time during which actuators are turned off, was introduced between each run to better understand the natural and unconstrained dynamics affecting the building. For this reason, acquisitions 2 and 3 feature different run lengths and downtime. While this does not directly affect the quality of the data, it does mean that phases 2 and 3 exhibit different run structures compared to the other acquisitions.

## Ethics Statement

The authors confirm having read and followed the ethical requirements for publication in Data in Brief and confirm that the current work does not involve human subjects, or any data collected from social media platforms.

## CRediT Author Statement

**Alessandro NOCENTE:** Methodology, Data Curation, Writing – Review and Editing **Odne Andreas OKSAVIK:** Software, Resources **Massimiliano RUOCCO:** Conceptualization, Methodology, Supervision, Writing – Review and Editing **Zachari THIRY:** Methodology, Data Curation, Formal Analysis, Writing – Original Draft

## Data Availability

ZenodoThe RICO Dataset (Original data).
